# MicroRNAs as a Potential New Preventive Approach in the Transition from Asymptomatic to Symptomatic Multiple Myeloma Disease

**DOI:** 10.3390/cancers13153650

**Published:** 2021-07-21

**Authors:** Vanessa Desantis, Antonio Giovanni Solimando, Ilaria Saltarella, Antonio Sacco, Viviana Giustini, Marta Bento, Aurelia Lamanuzzi, Assunta Melaccio, Maria Antonia Frassanito, Angelo Paradiso, Monica Montagnani, Angelo Vacca, Aldo M. Roccaro

**Affiliations:** 1 Unit of Internal Medicine and Clinical Oncology, Department of Biomedical Sciences and Human Oncology, University of Bari “Aldo Moro”, 70124 Bari, Italy; vanessa.desantis@uniba.it (V.D.); antonio.solimando@uniba.it (A.G.S.); ilaria.saltarella@libero.it (I.S.); aurelia.lamanuzzi@libero.it (A.L.); assuntamel@hotmail.it (A.M.); 2 Department of Biomedical Sciences and Human Oncology, Pharmacology Section, Medical School, University of Bari “Aldo Moro”, 70124 Bari, Italy; monica.montagnani@uniba.it; 3 IRCCS Istituto Tumori “Giovanni Paolo II” of Bari, 70124 Bari, Italy; a.paradiso@oncologico.bari.it; 4 Clinical Research Development and Phase I Unit, ASST Spedali Civili di Brescia, 25123 Brescia, Italy; saccoantonio74@gmail.com (A.S.); viviana.giustini@gmail.com (V.G.); 5 Centro Hospitalar Lisboa Norte, Department of Hematology and Transplantation, Institute of Molecular Medicine, University of Lisbon, 1649-035 Lisbon, Portugal; marta.leal.bento@campus.ul.pt; 6 Unit of General Pathology, Department of Biomedical Sciences and Human Oncology, University of Bari “Aldo Moro”, 70124 Bari, Italy; antofrassanito@gmail.com

**Keywords:** microRNAs, miRNA-based therapy, myelomagenesis, monoclonal gammopathy of undetermined significance, multiple myeloma

## Abstract

**Simple Summary:**

Multiple myeloma (MM) is the second most common haematologic malignancy, and it remains an incurable disease despite the advances of novel therapies. It is characterised by a multistep process that arises from a pre-malignant asymptomatic status-defined monoclonal gammopathy of undetermined significance (MGUS), evolves to a middle stage named smouldering myeloma phase (SMM), and culminates in the active disease (MM). Identification of early and non-invasive markers of the disease progression is currently an active field of investigation. In this review, we discuss the role and significance of microRNAs (miRNAs) as potential diagnostic biomarkers to predict the clinical transition from MGUS/SMM status to MM.

**Abstract:**

Multiple myeloma (MM) is a hematological malignancy characterised by proliferation of clonal plasma cells (PCs) within the bone marrow (BM). Myelomagenesis is a multi-step process which goes from an asymptomatic phase, defined as monoclonal gammopathy of undetermined significance (MGUS), to a smouldering myeloma (SMM) stage, to a final active MM disease, characterised by hypercalcemia, renal failure, bone lesions anemia, and higher risk of infections. Overall, microRNAs (miRNAs) have shown to significantly impact on MM tumorigenesis, as a result of miRNA-dependent modulation of genes involved in pathways known to be crucial for MM pathogenesis and disease progression. We aim to revise the literature related to the role of miRNAs as potential diagnostic and prognostic biomarkers, thus highlighting their key role as novel players within the field of MM and related premalignant conditions.

## 1. Introduction

Multiple myeloma (MM) is a plasma cells (PCs) dyscrasia, characterised by clonal expansion of PCs within the bone marrow (BM), coupled with a spectrum of clinical and biological features, i.e., hypercalcemia, renal failure, and bone lesions [1]. Myelomagenesis is a multistep process [2] in which MM arises from a pre-malignant asymptomatic status defined as monoclonal gammopathy of undetermined significance (MGUS) [3]. MGUS is present in >3% of the population; it remains stable, and no therapy is needed for years. It may otherwise evolve to MM with a rate of 1% per year [4]. While MGUS individuals are usually asymptomatic, they can have enhanced morbidity and mortality over the general population [5], most likely due to a significantly enhanced risk of malignant transformation, comorbidities [6], and infections [7], similarly to the full-blown disease phenotype [8,9]. Clearly, these findings warrant further translational investigation to pursue clinical management of the precursor states of MM, e.g., through the optimal combination of strategies that aim to halt progression to MM, in order to more efficiently sketch patients deserving preventive or curative approaches.

Noteworthily, MGUS is typically followed by the smouldering myeloma (SMM) phase that represents a middle stage between MGUS and MM, and that is characterised by 10–60% clonal BM PCs, and urinary and/or serum M protein at levels more than 500 mg/24 h and 30 g/L, respectively [10]. The SMM progression risk to evolve into MM is approximately 10% per year [11]. To date, no factors have been identified as biomarkers that could help to predict the transition from MGUS/SMM to MM. Recent studies point to microRNAs (miRNAs or miR) as new prognostic and diagnostic players in MM pathogenesis and progression [12,13,14]. miRNAs deregulation is associated with tumour initiation and progression as well as with PCs differentiation and myelomagenesis [15].

miRNAs are small single-stranded RNA molecules between 19 and 25 nucleotides in length. They regulate gene expression at post-transcriptional levels by binding to specific sites within the 3′ untranslated (3′-UTR) region of the mRNA targets, in order to determine either translation or inhibition [16]. miRNAs biogenesis begins in the nucleus, where they are transcribed by the RNA polymerase II into polyadenylated and capped pri-miRNAs [17,18]. The pri-miRNAs hairpin-shaped stem-loops are cleaved by the Drosha RNase III endonuclease forming pre-miRNAs. The latter are transported into the cytoplasm by Exportin-5/RanGTP and then converted into short double-stranded miRNAs duplex (miRNA:miRNA*) by Dicer, a ribonuclease-III [19]. The mature miRNAs, associated with Argonaute (AGO) 2 protein, bind to the RNA-induced silencing complex (RISC), while miRNA* is discarded. The single-stranded miRNAs connect to mRNA target 3′UTR and lead to degradation of the target mRNA transcripts, resulting in the inhibition of translation and/or to induction of deadenylation and mRNA decay [20,21].

Aberrant miRNAs expression regulates different cellular processes, i.e., apoptosis, proliferation, differentiation, immune response, metabolism, invasion, migration, and drug resistance [22,23,24,25]. Specifically, a single miRNA may act on different target gene transcripts, inducing several effects on gene expression, therefore dysregulated miRNAs in MM could act as oncogenes or tumour suppressors [15,26]. Different miRNAs have been studied in MM and in its precursor states of MGUS/SMM, revealing the miRNA critical role in normal PCs development and in the early stages of myelomagenesis [15].

The miRNAs are released within the body fluids, i.e., peripheral blood (PB), serum, and plasma. Usually, miRNAs levels are stable in PB, but they may vary depending on serum and/or to plasma detection in relation to their release from platelets or white blood cells: indeed, serum samples have increased levels of extracellular miRNAs over the plasma ones [27]. Specifically, a miRNA biomarker shed into PB could be altered in both serum and plasma [12] in association to different players present into the BM microenvironment (BMME), i.e., cytokines, growth factors and chemokines transferred via exosomes in the recipient cells [28,29,30]. Nowadays, several studies have highlighted the role of exosomes, as small extracellular vesicles (EVs) involved into the mechanisms of cell-to-cell communication [31,32,33]. Exosomes transport growth factors, proteins, miRNAs, and mRNA toward target cells and they can be secreted by MM cells, thus modifying the BMME that regulates cancer cells responses and promotes MM progression [10,34,35].

This review focuses on miRNAs as potential diagnostic biomarkers involved in the transition from MGUS/SMM to MM. We also considered the serum biochemical parameters, i.e., hemoglobin (Hb), thrombocytes (PLT), albumin (ALB), M-protein, β_2_-microglobulin (β_2_-MG), creatinine (Cr), and C-reactive protein (CRP) that correlate with miRNAs levels in MM progression [36]. Analysis of miRNAs may support the development for a new clinical management of MGUS and SMM patients with the introduction of innovative and curative approaches to prevent progression to MM.

## 2. miRNA Differential Expression during Myelomagenesis

**Figure 1:**
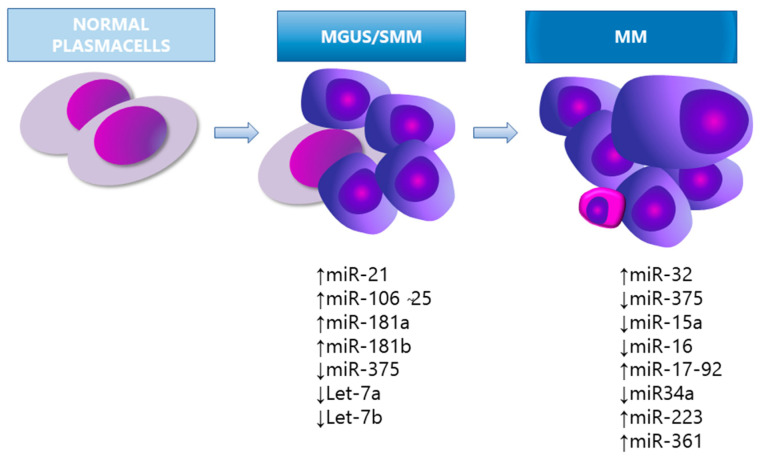
miRNA expression in the pathogenesis of MM. MGUS, monoclonal gammopathy of undetermined significance; SMM, smouldering myeloma.

miRNAs play a key role in oncogenesis through post-transcriptional gene regulation that influences the proliferation, differentiation, apoptosis, metastasis, and survival of cancer cells. During carcinogenesis, the changes responsible for deletion or amplification of entire chromosomal regions, occurring within miRNA coding loci, may cause a decrease or increase in the expression of the given miRNA, respectively. In particular, miRNA downregulation may target oncogenes, thus resulting in silencing of the related expression and function. In parallel, overexpressed miRNAs may contribute to carcinogenesis by inhibiting the activity of tumour suppressor genes. Despite the limited number of studies on the role of miRNAs in MM and MGUS, several evidences uncovered miRNAs as pivotal regulators of myelomagenesis [15] (Figure 1).

Pichiorri et al. [37] examined the different expression profiles of miRNAs in selected PCs of healthy donors (HD) versus MGUS and MM patients. The studies show that miRNA-21, -181a, 181-b, and the miRNA-106b-25 cluster (specifically miRNA-93.25 and -106b) were significantly overexpressed in MGUS compared to HD [37]. The miRNA-21, -18a and the miR106b-25 cluster were also overexpressed in MM. Authors suggested that the differently expressed miRNAs boost the development of the abnormal PCs population into MGUS and MM.

In vitro and in vivo data on MM cell lines show that miRNA-181a, -181b were upregulated, leading to enhanced MM tumour growth, as a result of modulation of cell proliferation, cell cycle progression, apoptosis, and cell invasion [37]. In association with miRNA-106b, miRNA-181a and b bind to the 3′UTR of the P300/CBP-associated factor (PCAF), a positive regulator of p53 that is highly deregulated in MM disease [37]. PCAF is a histone acetyltransferase that positively regulates p53 through acetylation, suggesting the potential role of the above-mentioned miRNAs on the alteration of the p53 pathway [37]. This supports the evidence that the dysregulation of the p53 pathway is involved in MM pathogenesis and, in particular, in drug resistance [38].

Moreover, the miRNA-21 and miRNA-106b-25 cluster may trigger secondary events that eventually lead to full-blown disease, by targeting PTEN, BIM, and p21, which are tumour suppressants that inhibit survival and promote cell apoptosis in MM pathogenesis [16]. The differential miRNAs expression in myelomagenesis was investigated by Wang et al. [12] in 17 patients with MGUS, 17 with SMM, 13 with MM, and 12 HD. Four out of eleven miRNAs were significantly decreased in patients with MGUS (let-7i, miRNA-15a, -16 and -106b), suggesting that aberrant miRNAs expression may be associated with early events in PC neoplasia, and potentially contribute to MGUS transition towards SMM [12]. Eight out of eleven miRNAs were decreased in patients with SMM (let-7a, let-7b, let-7i, miRNA-15a, -15b, -16, -106b and -20a) and three miRNAs (miR-21, miR-223, and miR-361) were significantly decreased in MM but not in MGUS/SMM patients, suggesting that downregulation of this group of miRNAs may be related to later events during disease progression [12]. The let-7a and let-7b miRNA regulate MYC gene expression and pinpoint the potential role of miRNA-induced epigenetic regulation of MYC as an additional driver of myelomagenesis [12]. Indeed, MYC is considered a key regulator of MM and its role in the progression of MGUS to MM was first established in a MYC-driven MM transgenic mouse model, called Vk*MYC [39].

Moreover, miRNA-15 and -16 downregulation may represent loss of oncosuppressors by evading the AKT kinase, the ribosomal protein S6, the MAP kinases (MAPK), and MAP3K-inositol trisphosphate (IP3) brake [12,40]. Collectively, the authors propose miRNAs as biomarkers for diagnosis, progression, and prognosis of the MM precursors stages [12], being a suitable target of non-invasive tests in human serum and plasma, carried by EVs linked to proteins such as nucleophosmin or AGO2. This hypothesis stimulated further validation comparing PB samples derived from MM and MGUS patients. Compelling data identified a differently expressed miRNAs basket, in which miRNA-720 and downregulation of miRNA-1308, -5011-5p, -6499-3p, -6722-5p, and -6849-3p occurred [13,14] (Table 1). The pathways analysis confirmed the role of miRNAs in oncogenic signaling [36].

## 3. Circulating miRNAs as Biomarkers in MM Progression

Cell-free miRNAs in the serum, plasma, saliva, urine, and other body fluids bind to proteins and are protected by vesicles, including exosomes, lipid particles, and apoptosis bodies to avoid degradation [30,47], inducing different effects on target tumour cells and prompting cancer outcomes [48]. The way through which circulating miRNAs are secreted in the tumour microenvironment is unclear. Valadi et al. [49] hypothesise that miRNA overexpression is associated with the high rate of proliferation and cell lysis in tumours, whereas another study suggests that circulating miRNAs derive from immunocytes at the tumour microenvironment [50]. Overall, these findings may provide the rational for considering miRNAs as potential MM biomarkers [3]. Indeed, a different circulating miRNAs profile is associated with MM patients compared to HD, MGUS, and SMM patients.

A broad systematic review of the available literature data was performed by Peipei Xu et al. [51] to investigate the miRNAs relevance that can be assessed in envisaging the clinical outcome of subjects suffering from MM. While different miRNAs were uncovered to be associated with the diverse overall survival (OS) and progression free survival (PFS) in MM, seven miRNAs emerged as the most relevant from the published studies dealing with MM. Specifically, miRNA-15a, -16, -25, -744, and let-7e were associated with dismal OS. Decreased miR-15a, -16, and -25, and enhanced -92a, levels, correlated with worse PFS.

Since MGUS represents an asymptomatic pre-malignant stage, it is essential for the clinicians to identify biomarkers predicting the risk for progression to active MM. Jones et al. [13] found that the combination between miRNA-720 and -1308 enables to distinguish HD from MM patients; additionally, the association of miRNA-1246 with -1308 discriminates MGUS from the MM condition.

Kubiczkova et al. [14] demonstrated that the aberrant expression patterns of five circulating miRNAs (miRNA-744, -130a, -34a, let-7d, and let-7e) are relevant in MGUS and MM patients compared to HD. Indeed, the combined deregulation of miRNA-34a and let-7e may help discriminating MGUS and MM patients from HD while the lower levels of miRNA-744 and let-7e indicate shorter OS in MM patients [14]. Interestingly, levels of miRNA-34a appear to follow a specific profile: while downregulated in several other cancer types [52,53,54] and in MGUS, miRNA-34a overexpression has been reported in active MM in association to the inhibition of cell proliferation, colony formation, and increased cancer stem cells (CSCs) apoptosis in vitro, via a mechanism that reduces the expression of the transforming growth interaction factor 2 (TGIF2) [41]. Despite some controversies still incompletely clarified, these findings underline the complex role of miRNA-34a. On the same line, the significant anti-proliferative activity, apoptotic effects, and modulation of gene expression observed with synthetic miR-34a mimics suggest that miRNA-34a is likely deregulated in MM [55]. Recent studies show that miRNAs are important cargos transferred by circulating exosomes and that they can be useful as prognostic biomarkers to predict disease progression risk and response to therapy [10]. Zhang et al. [56] revealed that circulating levels of exosomal miRNAs were significantly different in HD compared to SMM and MM patients.

Manier et al. [57] showed how MM patients’ exosome-derived circulating miRNA-18a and let-7b were associated with poor outcomes, in terms of OS and PFS, once more reinforcing the proposed use of circulating exosomal miRNAs to identify patients with MM and to distinguish those with poor outcomes [57].

To support the idea that the communication between cancer stromal cells (SCs) and PCs is a key factor in MM pathogenesis and that a crucial role in cell-to-cell communication is associated with exosomes, Frassanito et al. [58] demonstrated that upregulation of exosomal miRNA-214 and -27 is related to the transition from MGUS to MM stage. The miRNA-214 targets PTEN, that is, the main negative regulator of the PI3K/AKT pathway for MM cells proliferation, apoptosis, invasion, and metabolism [59]. Authors also showed a reciprocal communication between fibroblasts and PCs via exosomes as a key factor in MM pathogenesis. Specifically, via an exosome-mediated release, MM PCs exosomes transform resident fibroblasts into cancer-associated fibroblasts (CAFs) that over-express miRNA-214 and -27b; these CAFs, in turn, support the growth of PCs and MM progression [58]. Hao et al. [60] demonstrated that the overexpression of serum miRNA-214 indicates a poor outcome in MM patients, since its high expression levels were associated with shorter PFS and OS. Additionally, MM patients with high serum levels of miRNA-214 have a shorter PFS and OS compared to patients with low expression levels.

In this perspective, the association between expression levels of miRNAs and the life expectancy of MM patients seems to achieve relevance, and strictly depends on the nature of specific miRNAs. For example, the over-expression of miRNA-20 and -148a plasma levels in MM correlate with relapse-free survival rate [61]; moreover, elevated miRNA-25, -16, and -30a levels in the serum of MM patients correlate with a longer OS as compared to MM patients with lower serum levels of the same miRNAs [62]. Thus, the expression levels of miRNAs may be a new field of research to predict life expectancy in MM patients.

Within the context of the BM niche, BM endothelial cells (BMECs) and endothelial progenitor cells have been shown to play a crucial role in MM pathogenesis [63,64,65,66,67,68]. The interactions between MM cells and the components of the microenvironment exert their regulatory effect on cancer cells via miRNAs. For instance, BMECs have been shown to be actively involved in nursing MM cells in their milieu, by creating a permissive immune infiltrate [68] and representing a potential theragnostic druggable target [67,69,70]. It is now clear that the MM milieu is hypoxic, and that hypoxia promotes angiogenesis, invasion, and tumour progression. Hypoxia also regulates the expression of miRNAs in cancer cells: in particular, the reduced expression of miRNA-199a-5p in turn targets the hypoxia-inducible transcription factor (HIF-1-alpha) overexpressed in MM cells [71], pointing towards a potential Achilles’ heel of MM that might be exploited therapeutically by inhibiting hypoxia, HIFs, and mTOR [72,73,74]. The interaction between cancer cells and stromal cells also determines the production of various pro-angiogenic factors such as VEGF. Roccaro et al. [73] have identified several deregulated miRNAs in MM cells; among them, miRNA-15a/16 have been uncovered to be significantly decreased, therefore helping to explain the impaired control in VEGF production by MM cells. Wu et al. [75] also described the role of AGO2 as a promoter of angiogenesis, acting through the overexpression of proangiogenic miRNAs such as the let-7 and miRNA-92a family and the downregulation of antiangiogenic miRNA-45. The let-7 family regulates VEGF levels and promotes angiogenesis via the reduction of HIF-3alpha, negative regulator of the HIF pathway in endothelial cells. miRNA-92a, overexpressed in MM cells, regulates VEGF levels and promotes angiogenesis. The miRNA-29 family comprises other members that act as a tumour suppressor. In particular, miRNA-29b favors the demethylation of the SOCS-1 promoter, which is hypermethylated in MM, and is also implicated in the reduced concentrations of IL-8, which promotes cell migration. Therefore, miRNA-29b negatively regulates the migration of MM cells and endothelial cells [64]. Circulating miRNAs released into exosomes by cancer cells, stromal and endothelial cells feed into a vicious cycle into the MM niche [63]. The involvement of miRNAs in exosomes might be implicated in both in promoting tumour activity as well as in favoring the angiogenic switch mediated by MM cells. Thus, approaches combining anti-angiogenic therapy and immunotherapy via miRNAs mimicking could improve therapeutic response by hijacking fueling mechanisms feeding an abnormal stromal and endothelial cell behaviors that sustain aberrant angiogenesis [71,76,77].

To confirm that deregulated miRNAs change their expression profile during MM progression, Yyusnita et al. [78] demonstrated that miRNA-494, -130a, let-7i, and let-7c are expressed in MM patients only at diagnosis, while miRNA-148a, -1225, -423, miRNA-484, -99a, -106a, -224, -638, and let-7b are expressed only during the follow-up. On the other hand, miRNA-19a and -4254 may better characterise MM patients in remission, being lower in MM patients presenting with a relapse disease [79].

Overall, the interrelated observations from multiple studies support the view that exosomal miRNAs may serve as candidate-circulating markers to monitor tumour progression and activity in MM patients.

## 4. Correlation between Circulating miRNA and Biochemical Parameters in MM Patients

Several factors, including hematological profile, physical evaluation, BM examination, serum electrophoresis patterns, clinical symptoms, medical history, and biochemical parameters, are conventionally taken into consideration for an accurate MM diagnosis [80].

Reportedly, the alteration of biochemical parameters including Hb, M-protein, lactic acid dehydrogenase (LDH), PLT, ALB, β2-MG, and CRP in combination with the deregulated profile of circulating miRNAs expression is estimated to predict MM progression and patient outcome [14,36,48] (Table 2). This type of association may be useful to distinguish the MM activity phases, to provide an early treatment, and to improve the disease prognosis. Zhang et al. [48] demonstrated a positive correlation of miRNA-744, -130a, let-7d, and let-7e expression levels with Hb and thrombocyte counts; conversely, the same miRNAs were found to be negatively correlated with β_2_-microglobulin levels. A significant negative correlation was measured when Hb and ALB were associated with miRNA-34a [48]. Only three of the considered miRNAs (miRNA-744, let-7d and let-7e) were found positively associated with ALB, while let-7 negatively correlated with the level of monoclonal immunoglobulin [14]. miRNA-202 correlates positively with β_2_-MG and light chain, while it correlates negatively with LDH and light chain levels [81].

As mentioned above, serum biochemical parameters show significant differences during MM progression, and their relationship with differentially expressed miRNAs may help to establish an early diagnosis. Li et al. [36] found that miRNA-134-5p, -107 and -15a-5p are upregulated in MGUS and MM patients, and that miRNA-134-5p and -107 negatively correlate with LDH in MM and with Hb in MGUS patients. Overall, the combination of miRNA-107 and -15a-5p with Hb levels was regarded as a potential diagnostic biomarker to distinguish MM from MGUS patients [36]. Of striking difference appears the overexpression of miRNA-92a in plasma levels from MGUS compared to MM patients, while no significant changes are detected between SMM and MM patients [82]. This suggests that levels of miRNA-92a might be helpful to monitor the therapeutic response and to check the MM progression [82].

A close relationship between MM clinical symptoms and abnormal expression of miRNAs has not been detected. However, it is known that IL-6 is a direct target gene of let-7 miRNA, whose downregulation is associated with IL-6 overexpression [83]. IL-6 plays relevant roles in MM (i.e., growth factor, regulation of MM cell proliferation, modulation of MM cell death) and its elevated serum levels closely correlate with poor prognosis and short OS [84]. In the same study, miRNA-185-5p levels were positively correlated with Hb, while let-7c-5p, miRNA-140-3p, -185-5p, and -425-5p negatively correlated with Cr and IL-6 levels. β-CTX, which is an indicator of osteoclasts activity, showed negative correlation with exosomal miRNAs let-7c-5p, miRNA-140-3p, -185-5p, and A-425-5p [85]. Overall, all detected miRNAs were found to be negatively associated with disease progression as well as indicators of tumour burden, such as β2-MG and load of myeloma PCs [85].

Altogether, the data described above suggest that in MM patients, the deregulated expression levels of miRNAs are closely related to those biochemical parameters indicating the development and progression of MM disease.

Of equal interest is the correlation between the expression of miRNAs and different cellular pathways involved in MM progression. For instance, Zhang et al. [56] have proposed serum exosomal microRNAs as novel and independent biomarkers for MM. A different expression in serum exosomal miRNAs can be detected in smouldering myeloma (SMM) or in active MM patients compared to healthy individuals. For seven miRNAs, including let-7c-5p, miR-20a-5p, -103a-3p, -140-3p, -185-5p, -425-5p, -4505, and -4741, expression level is lower than in healthy individuals, suggesting the potential association between a decreased expression levels of exosomal miRNAs and the disease progression of MM. Of note, the let-7 family is associated with MYC overexpression, which might promote progression from MGUS to MM [86,87] and is correlated with poor prognosis [2,88]. miR-20a promotes tumour cell proliferation and inhibits apoptosis [89]; miR-103 can reduce the phosphorylation of Yes-associated protein (YAP), which is a regulator of the Hippo pathway and plays an important role in cell proliferation and MM progression [90]. In addition, analysis of miRNAs in the SMM group revealed the abnormal expression of miR-140-3p, -185-5p, whereas miR-4741 expression has been associated with clinical manifestations of MM and potentially involved in MM malignant transformation. Recently, to identify the prognostic value of miRNAs in patients with MM, Xu et al. [51] performed a literature search and found that upregulated levels of miR-92a and downregulated expression of miR-16, miR-25, miR-744, miR-15a, let-7e, and miR19b were associated with poor prognosis in patients with MM.

For all the mentioned miRNAs, drawbacks should be taken into consideration, such as the limited number of studies available, the marked heterogeneity among the performed analyses, the differences in clinical patient features, and follow-up durations, as well as assay and miRNAs extraction methods, or cut-off values for miRNAs expression levels.

### 4.1. miRNA as New Clinical Biomarkers in MM Therapy

An essential step for MM diagnosis is represented by BM biopsy and BM aspirate, which are certainly considered as invasive procedures. Therefore, several advantages may come from using circulating miRNAs as sensitive MM biomarkers, since they can be collected in the PB through a minimally invasive procedure [13,14], and their expression profile should give important indications to identify clinical and therapeutic MM outcomes [48]. Moreover, integrated next-generation tools, such as single-cell RNA-seq, from a large cohort of MGUS and SMM, will functionally deconvolute MM heterogeneity and unravel determinants of disease progression, potentially delivering a paradigm for the use of molecularly driven precision medicine and support MM risk stratification.

Autologous stem cell transplant (ASCT) in MM patients is a recognised therapy whose efficacy may vary in each individual patient. In this context, the identification of circulating markers able to predict patients’ outcome after ASCT could be extremely useful. Navarro et al. [91] showed that patients undergoing ASCT, presenting with a high expression of serum miRNA-19b and -331, have significantly prolonged PFS.

Conventional therapeutic approaches include the use of immunomodulatory drugs, proteasome inhibitors, monoclonal antibodies, alkylating drugs, and steroids. However, MM patients reveal a heterogeneous response (remission rate) and, quite often, drug-resistance which, in turn, is responsible for a refractory MM condition [92]. Therefore, several studies have also investigated the relevance of miRNAs as potential markers of drug resistance. For instance, miRNA-29b has been shown to be significantly reduced in bortezomib-resistant MM cells, as well as in MM cells resistant to second-generation proteasome inhibitors (PI) such as carfilzomib and ixazomib. Specifically, Jagannathan et al. [93] demonstrated that the increased expression of miRNA-29b enhanced the bortezomib-induced accumulation of ubiquitinated proteins, without leading to enhanced autophagosome formation. As a consequence, the combination of miRNA-29b and bortezomib may decrease bortezomib-resistance, increasing the sensitivity of MM cells to the drug resulting in enhanced apoptosis and inhibition of the PI3K-AKT signaling pathway [94]. On the same line, Wang et al. [95] revealed that miRNA-21 in combination with bortezomib, dexamethasone, and doxorubicin had a synergistic effect in mediating MM cell apoptosis, as compared to the effect of each drug used as monotherapy.

Other studies underline the potential role of miRNAs in predicting response to therapy. Hao et al. [79] identified 95 dysregulated serum miRNAs in newly diagnosed MM patients and suggested that a combination of miRNA-19a and -4254 may distinguish MM from HD. In more detail, the reduced expression of miRNA-15a was shown to be of prognostic relevance, as it correlated with disease progression and poor prognosis [40]. Moreover, MM patients with low levels of miRNA-15a did not show an improvement in PFS and OS, thus suggesting the possible role of miRNA-15a in mediating drug resistance [40]. In parallel, other studies have reported the increased levels of serum exosome-derived miRNA-16-5p, -15a-5p, -20a-5p, and -17-5p in bortezomib-sensitive patients compared to bortezomib-resistant ones [96].

Steroids are often part of multi-drug-based treatments, based on the rational of high-dose steroid-induced MM cell apoptosis [97]. Palagani et al. [98] demonstrated that the combination between miRNA-150-5p synthetic vector, and low doses of steroids had a synergistic anti-MM effect. Of note, increased levels of the miRNA-221/222 family have been linked to enhanced survival of dexamethasone-resistant MM cells that might result, at least in part, by modulation of the PUMA-BAK-BAC pathway [99].

Gullà et al. [100] have suggested the existence of an inverse correlation between miRNA-221/222 expression and melphalan sensitivity. In vitro and in vivo studies have demonstrated how targeting miRNA-221/222 overcomes melphalan resistance and enhances MM cell apoptosis, even in the presence of the supportive BMME. The anti-MM effect was mediated, at least in part, by upregulation of pro-apoptotic BCL2 Binding Component 3 (BBC3)/PUMA protein, coupled with modulation of drug influx-efflux transporters Solute Carrier Family 7 Member 5 (SLC7A5)/LAT1 and the ATP-Binding cassette (ABC) transporter ABCC1/MRP1. Importantly, the use of the locked nucleic acid (LNA) anti-miRNA-221 could reverse melphalan resistance in in vivo models, thus providing the preclinical rationale for using miRNA-221-neutralising therapies in MM [100].

Finally, since miRNAs have shown to correlate with MM clinical outcome [101] and variable CD surface markers expression clusters [102], the dissection of this molecular crosstalk [101,102] holds the potential to identify a semipersonalised anti-neoplastic strategy, pointing towards both surface markers [103,104] and epigenetic mechanisms [10,73,105].

### 4.2. microRNA as a New Preventive Approach to Detect the Asymptomatic to Symptomatic MM Transition

It would be of crucial importance to consider the role of miRNAs as biomarkers detecting the asymptomatic to symptomatic MM transition, within the setting of a clinical trial. However, at the present, available results are still too preliminary to achieve statistical significance. Nevertheless, two paradigmatic examples aiming to deep-screen and prevent MM by collecting high-throughput data are represented by the researches performed by Pichiorri et al. [37] and within the iStopMM initiative (NCT03327597; NCT03815279) [106]. This last approach, taking advantage of a genetic screening in a population-based nationwide clinical trial with early intervention, offers the potential to collect a large biobanking repository available for future biological studies, including epigenetic profiling. Similarly, studies aimed to discriminate high-risk features in the premalignant phases [107,108,109] and the SWOG S0120 [110] are expected to provide further insights into the molecular mechanisms of plasma cell dyscrasias. To date, statistically powered prospective studies stemming from the existing established experience [62] are still lacking, and represent a partially unmet need in order to standardise the progression of MGUS or SMM toward overt full-blown MM.

## 5. Conclusions

Collectively, circulating miRNAs may represent novel biomarkers in MM. They can be obtained through non-invasive procedures. They have been indicated as a valuable resource for better defining MM pathogenesis, diagnosis, and prognosis. In perspective, miRNAs may become novel therapeutic tools for either silencing and/or over-expressing genes in MM cases where the given miRNAs are upregulated or downregulated, respectively. Nevertheless, further long-term clinical practices are still required and essential before considering miRNA-based therapy as the routine approach in the MM clinical management. Indeed, additional comprehensive and detailed studies are needed to understand the molecular mechanisms able to explain the multiple effects of miRNAs in MM. Hopefully, ongoing and future analyses of several RNA signatures will allow more extensive characterisation of biology and prognosis with respect to individual, personalised, and innovative therapies.

## Figures and Tables

**Table 1:** miRNAs involved in myeloma pathogenesis and their functions.

miRNA	miRNA Modulation	Effect on Myeloma Pathogenesis	Target	References
Let-7a Let-7b	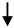	Related to disease initiation, progression	MYC in t(11;14)	[12]
miRNA-34a	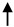 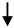	Inhibited cell proliferation, colony formation, and increased cancer stem cells apoptosis	TGIF2	[14,41]
miRNA-15a/ miRNA 16	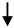	Enhanced proliferation in MM	AKT3, ribosomal protein S6, MAPK, NF-kB, MAP3K1	[12,40]
miRNA-21	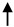	Enhanced cell survival	STAT3 PTEN, BIM and p21	[34] [37]
miRNA-134-5p, miRNA-107, miRNA 15a-5	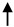	Enhanced cell proliferation in MGUS and MM	ITGB1, PIK3R1	[36]
miRNA-17-92	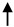	Decreased apoptosis in MM	PTEN, E2F1, Bcl2/ BIM	[37]
miRNA-181 a/b	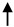	Enhanced cell proliferation, cell cycle, apoptosis and invasion in MM	p53, PCAF, NOVA1	[37,42]
miRNA-375	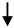	Enhanced cell survival and proliferation both in MGUS and MM	PDK1	[43]
miRNA-19a, miRNA 19b	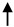	Increased, constitutive JAK/STAT3 activation	SOCS-1	[44]
miRNA-32	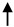	p53 function interference in MM	PCAF	[45]
miRNA106-b, miRNA-93, miRNA-25	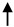	Increased in MGUS and MM; enhanced cell survival	PCAF	[46]

**Table 2:** Correlation between MM biochemical parameters and circulating miRNAs.

Biochemical Parameter	miRNA	Parameter/miRNA Correlation	MM Correlation	Ref.
M-protein	miRNA-1246	Negative	MM vs. HD	[13]
C-reactive protein (CRP)	miRNA-744	Negative	MM vs. HD	[14]
	let-7e	Negative		
Albumin (ALB)	let-7d	Positive	MM vs. HD MM vs. HD	[14] [48]
	let-7e	Positive		
	miRNA-34a	Negative		
	miRNA-744	Positive		
lactic acid dehydrogenase (LDH)	miRNA-202	Negative	MM vs. HD MM vs. HD MM vs. HD	[36,81]
	miRNA-34a-5p	Negative		
	miRNA-107	Negative		
Thrombocyte counts	miRNA-744	Positive	MM vs. HD	[48]
	miRNA-130a	Positive		
	let-7d	Positive		
	let-7e	Positive		
β_2_-microglobulin (β_2_-MG)	miRNA-744	Negative		
	miRNA-130a	Negative		
	let-7d	Negative		
	let-7e	Negative		
	miRNA-202	Positive		
	let-7c-5p	Negative		
	miRNA-140-3p	Negative		
	miRNA-185-5p	Negative		
	miRNA-425-5p	Negative		
Creatinine (Cr)	miRNA-744	Negative	MM vs. HD MM vs. HD	[48] [85]
	miRNA-130a	Negative		
	let-7d	Negative		
	let-7e	Negative		
	let-7c-5p	Negative		
	miRNA-140-3p	Negative		
	miRNA-185-5p	Negative		
	miRNA-425-5p	Negative		
Kappa light chain	miRNA-202	Positive	MM vs. HD	[81]
Lambda light chain	miRNA-202	Negative	MM vs. HD	[81]
IL-6	let-7	Negative	MM vs. HD	[85]
	let-7c-5p	Negative		
	miRNA-140-3p	Negative		
	miRNA-185-5p	Negative		
	miRNA-425-5p	Negative		
β-CTX	let-7c-5p	Negative	MM vs. HD	[85]
	miRNA-140-3p	Negative		
	miRNA-185-5p	Negative		
	miRNA-425-5p	Negative		
